# Identification of New Potential Biotherapeutics from Human Gut Microbiota-Derived Bacteria

**DOI:** 10.3390/microorganisms9030565

**Published:** 2021-03-09

**Authors:** Bernardo Cuffaro, Aka L. W. Assohoun, Denise Boutillier, Véronique Peucelle, Jérémy Desramaut, Samira Boudebbouze, Mikael Croyal, Anne-Judith Waligora-Dupriet, Moez Rhimi, Corinne Grangette, Emmanuelle Maguin

**Affiliations:** 1 U1019-UMR 9017-CIIL-Centre d′Infection et d′Immunité de Lille, Institut Pasteur de Lille, CNRS, Inserm, CHU Lille, Université de Lille, 59000 Lille, France; bernardo.cuffaro@gmail.com (B.C.); denise.boutillier@orange.fr (D.B.); veronique.peucelle@pasteur-lille.fr (V.P.); jeremy.desramaut@pasteur-lille.fr (J.D.); 2 Institut Micalis, MIHA Team, Université Paris-Saclay, INRAE, AgroParisTech, 78350 Jouy-en-Josas, France; landryassohoun@gmail.com (A.L.W.A.); samira.boudebbouze@inrae.fr (S.B.); moez.rhimi@inrae.fr (M.R.); 3 Laboratoire de Biotechnologie et Microbiologie des Aliments, UFR en Sciences et Technologies des Aliments, Université Nangui Abrogoua, Abidjan 00225, Côte d’Ivoire; 4 CRNH-O Mass Spectrometry Core Facility, 44000 Nantes, France; Mikael.Croyal@univ-nantes.fr; 5 Faculté de Pharmacie de Paris, UMR-S1139 INSERM, Université de Paris, 75006 Paris, France; anne-judith.waligora@u-paris.fr

**Keywords:** microbiota, microbiome, ecosystem, holobiont, live biotherapeutic products (LBP), next generation probiotics (NGP), functional screening, IBD, obesity

## Abstract

The role of the gut microbiota in health and disease is well recognized and the microbiota dysbiosis observed in many chronic diseases became a new therapeutic target. The challenge is to get a better insight into the functionality of commensal bacteria and to use this knowledge to select live biotherapeutics as new preventive or therapeutic products. In this study, we set up a screening approach to evaluate the functional capacities of a set of 21 strains isolated from the gut microbiota of neonates and adults. For this purpose, we selected key biological processes involved in the microbiome-host symbiosis and known to impact the host physiology i.e., the production of short-chain fatty acids and the ability to strengthen an epithelial barrier (Caco-2), to induce the release of the anti-inflammatory IL-10 cytokine after co-culture with human immune cells (PBMC) or to increase GLP-1 production from STC-1 endocrine cell line. This strategy highlighted fifteen strains exhibiting beneficial activities among which seven strains combined several of them. Interestingly, this work revealed for the first time a high prevalence of potential health-promoting functions among intestinal commensal strains and identified several appealing novel candidates for the management of chronic diseases, notably obesity and inflammatory bowel diseases.

## 1. Introduction

It is now widely accepted that the intestinal microbiome [1] plays a major role in our health. Indeed, the human host and its associated microbiome constitute a holobiont whose phenotype results from the combined expression of the host and associated microbiome genomes [2]. Better knowledge on the role of the host-microbiome symbiosis in human health and how perturbation of this homeostasis induces a shift from healthy to disease states remains burning questions in the field of holobiont and human health research [3,4].

Among others, the intestinal microbiome has a privileged position. At first, the fecal human microbiome is nowadays among the best-characterized microbiomes thanks to several international programs [5,6], the development of high-throughput sequencing techniques [7] allowing access to the quantitative composition of the dominant gut bacteria [8,9] and culturomics [10] to isolate and study previously unknown microorganisms. Second, numerous studies established that the gut microbiota is involved in intestinal maturation and homeostasis through numerous functions and is in a constant symbiosis and cross-talk with our human cells and organs [11]. For instance, colonization of our gut is crucial for the maturation and education of our digestive tract and cognate immune [12,13] and nervous systems [14,15] and its protection through the ecosystems barrier effect [16]. It plays also an important role in the production of essential compounds for our body such as vitamins [17], the maintenance of metabolic homeostasis [18], and even the healthy functioning of distal organs such as the brain [19], liver [20], and lung [21]. The gut microbiota is a highly dynamic ecosystem since its composition is for the most part, different for each human individual and influenced by age, geographical location, diet, and medication during the course of life. *Bacteroidetes* and *Firmicutes* are the most prevalent phyla in adults, together with *Proteobacteria* and *Actinobacteria* [7]. Although metagenomics has revolutionized the perception of the human microbiome and metabolomic analyses have contributed to the identification of derived metabolites acting on host cells, it remains important to better differentiate the strain-specificity among species and to decipher the functional properties of the bacterial community. Some correlation studies, comparing the microbiomes of patients and healthy individuals highlighted the association between dysbiosis and many chronic non-communicable diseases, notably metabolic syndrome, obesity [22,23], diabetes [24], inflammatory bowel diseases [25,26], liver diseases [27,28], and neurodegenerative and psychiatric disorders [29,30]. These works concluded on the importance of the diversity of the intestinal microbiota and a sustainable cross-talk between the microbiota and its host for healthy status. They also pinpointed specific species, strains, or metabolites signatures considered important for health. This allowed the selection of a limited number of bacteria considered to have clinical importance and potential health beneficial properties, such as *Faecalibacterium prausnitzii* [31], *Akkermentia muciniphila* [32], and *Eubacterium hallii* recently reclassified as *Anaerobutyricum hallii* or *A. soenhgenii* [33]. The functionality of these species as live biotherapeutic products (LBPs) have been assessed in experimental models [34,35,36] and started to be evaluated in clinical trials [37]. *Bacteroides* have been shown to be decreased in obese patients and the protective effect of selected strains has been shown in animal models [38,39]. We and others have also highlighted the probiotic potential of *Parabacteroides distasonis* [40,41].

While extensive screening have been performed for traditional probiotics, notably for bifidobacteria and lactobacilli, to identify the effective strains [42], such studies remain scarce for LBPs. Randomized controlled trials have reported the efficacy of some of these probiotic strains in different pathologies, such as IBD and obesity (for review see [43,44,45]) but remain scarce and controversial. This low effectiveness provided a rationale for the use of bacteria isolated from the gut microbiota as a source of Next Generation Probiotics (NGP) for the prevention or treatment of chronic diseases associated with microbiota dysbiosis. However, the functional characterization of most of the commensal strains residing in the gut remains in its infancy and calls for more studies leading to the identification of new strains with high health beneficial potentials. 

In this work, we investigated 21 commensal strains belonging to prevalent bacterial members of the human intestinal microbiota. To reach our objective, we combined different in vitro tests largely used for traditional probiotics [46,47], to assess their putative health-promoting properties, notably for immunomodulation, epithelial barrier strengthening effect, and production of the GLP-1 incretin. We also unraveled their ability to resist gastric conditions and to produce short-chain fatty acids (SCFA), main metabolites derived from bacterial fermentation and key players in human health. Our work revealed a high prevalence of these properties among the tested strains suggesting that strains with health beneficial properties can be easily found among gut bacteria. 

## 2. Materials and Methods 

### 2.1. Bacterial Strains and Growth Conditions

A set of 20 human commensal strains was chosen from the gut bacteria culture collections of UMR1319 Micalis, MIHA team, and UMR-S1139 INSERM. In addition, *Anaerobutyricum soehngenii* (previously *E. hallii*) was bought from the DSMZ collection (Table 1). The taxonomic assignation of each strain was verified based on the Blast comparison of the strain sequence of the V3-V4 variable region of the *16S* ribosomal RNA with the NCBI *16S* ribosomal RNA sequences database (data not shown). These strains were cultured at 37 °C in a Freter anaerobic cabinet under controlled atmosphere (Bio 300, Air liquide, Fr) in Brain-Heart Infusion-Yeast extract-Hemin medium (BHI-YH) [48] except *A. soehngenii* grown in the DSMZ Medium 104 supplemented after autoclaving with 50 mL/L of clarified rumen juice (https://www.dsmz.de/microorganisms/medium/pdf/DSMZ_Medium104.pdf (accessed on 3 March 2021)). For in vitro tests, overnight cultures at OD_600nm_ between 0.8–1 were centrifuged at 6000 rpm for 15 min at room temperature, washed in 1 volume of phosphate-buffered saline (PBS, pH 7.2), and bacteria were suspended at final concentrations of 10^8^ to 10^10^ CFU/mL in PBS containing 25% glycerol. These bacterial suspensions were then removed from the anaerobic cabinet and immediately frozen in liquid nitrogen prior to storage at −80 °C. A numeration was performed to check the viability and measure the CFU/mL of the bacterial pellets.

Two strains were used as controls in the PBMC assay: *Lactobacillus acidophilus* NCFM (provided by Dupont-Danisco) was grown without shaking at 37 °C in De Man, Rogosa and Sharpe broth (MRS, Difco, Detroit, MI, USA) and *Bifidobacterium animalis* subsp. *lactis* BB12 (provided by G. Vinderola, INLAIN, Argentina) was grown in MRS supplemented with 0.05% L-cysteine-hydrochloride (Sigma, St-Louis, MO, USA) under anaerobiosis (GENbag anaer, Biomérieux, Marcy l’Etoile, France). After overnight culture, bacteria were washed twice in sterile PBS buffer (pH 7.2) and suspended to a final concentration of 2 × 10^9^ CFU/mL in PBS.

### 2.2. Measurement of SCFA Production by GC-MS

OD_600nm_ of overnight culture were measured then the cultures were centrifuged at 6000 rpm during 15 min and collected supernatants were kept at −20 °C. Measurement of short-chain fatty acids (SCFA) was performed as previously described [49] with slight modifications. A stock solution of SCFA metabolites (Sigma-Aldrich, Saint-Quentin-Fallavier, France) was prepared and serially diluted to get ten calibration solutions. A working solution of internal standards was prepared in 0.15 M sodium hydroxide to get the following final concentrations: 75 mmol/L of D3-acetate, 3.8 mmol/L of D5-propionate, 2.5 mmol/L of ^13^*C*-butyrate, and 0.5 mmol/L of D9-valerate (Sigma-Aldrich). Samples (100 µL) were dissolved in 200 µL of sodium hydroxide solution at 0.15 M (Sigma-Aldrich). Twenty microliters of the internal standard solution were added to samples and calibration solutions.

After addition of the standards, each sample was acidified with 5 µL of hydroxide chloride 37% (Sigma-Aldrich) and then extracted with 1.7 mL of diethyl ether (Biosolve, Dieuze, France). Samples were stirred gently for 1 h and then centrifuged 2 min (5000 rpm, 4 °C). The organic layers were transferred into 1.5 mL glass vials and SCFAs were derivatized with 20 µL of tert-butyldimethylsilyl imidazole (Sigma-Aldrich). Samples were incubated for 30 min at 60 °C before analysis. Samples were finally analyzed by gas chromatography–mass spectrometry (model 7890A-5975C; Agilent Technologies, Montpellier, France) using a 30 m × 0.25 mm × 0.25 µm capillary column (HP1-MS; Agilent Technologies). The temperature program started at 50 °C for 1 min, ramped to 90 °C at 5 °C/min and then up to 300 °C at 70 °C/min. Selected ion monitoring mode was used to measure SCFA concentrations with ions at 117 m/z (acetate), 120 m/z (D3-acetate), 131 m/z (propionate), 136 m/z (D5-propionate), 145 m/z (butyrate and isobutyrate), 146 m/z (^13^*C*-butyrate), 159 m/z (valerate), and 168 m/z (D9-valerate). To ease the comparison between the various bacterial strains, the results were expressed as the mean SCFA concentrations divided by the optical density of the culture used to harvest the supernatants ± SEM.

### 2.3. Tolerance to Gastric Stress

The survival of the strains in simulated gastric juice (SGF, Minekus et al. [50]) was measured as follows. Bacterial cultures were centrifuged (6000 rpm, 10 min at 4 °C), washed twice with PBS (pH 7.2), and suspended in 0.2 mL of PBS to obtain the equivalent of 10^8^ to 10^10^ CFU/mL. After thawing, 50 µL of the frozen bacterial suspension were added to 950 µL of simulated gastric fluid (SGF) at pH 3. Briefly, SGF was composed of KCl 6.9 mM, HCl 15.6 mM, KH_2_PO_4_ 0.9 mM, NaHCO_3_ 25 mM, NaCl 47.2 mM, MgCl_2_ 0.1mM, (NH_4_)2CO_3_ 0.5 mM and adjusted to pH 3 using HCl 1 M. Porcine pepsin (Sigma Aldrich, France) and CaCl_2_ were added at final concentrations of 2.000 U/mL and 0.075 mM, respectively. Static incubation was performed in anaerobic condition for 2 h at 37 °C and viable counts were determined every 30 min by plating dilutions of each bacterial sample. Results were calculated as the mean ratio of the colony-forming units (CFU/mL) at a given time point on the initial CFU/mL at T0 ± SEM.

### 2.4. Immunomodulation Assay

The experimental protocol was approved by our institution committees (Institut Pasteur de Lille, agreement N° DC 2013–2022), in accordance with relevant guidelines and regulations. Blood samples were obtained from five healthy informed donors upon approved agreement (signed consents) by authorized staff. Peripheral blood mononuclear cells (PBMCs) were isolated from the blood as described before [51]. Briefly, after Ficoll gradient centrifugation (GE Healthcare Bio-Sciences, Uppsala, Sweden), PBMCs were recovered at the interface, washed in RPMI-1640 medium (Gibco, Life Technologies, Ghent, Belgium), and adjusted to 2 × 10^6^ cells per mL in RPMI supplemented with gentamicin (150 µg/mL), glutamine (2 mM) and 10% heat-inactivated fetal calf serum (Gibco, Life Technologies, Grand Island, NE, USA).

PBMCs were stimulated with bacteria at a bacteria-to-cell ratio of 10:1 (or not, control medium). After 24 h of stimulation at 37 °C under 5% CO_2_ atmosphere, the supernatants were collected, clarified by centrifugation (10 min at 1500× *g*), and stored at −20 °C until cytokine (IL-10, IL-12, IFNγ) measurements, performed with R&D Duoset ELISA kits (R&D, Minneapolis, MN, USA). *Lactobacillus acidophilus* NCFM was included as a pro-Th1 reference strain [34] and *Bifidobacterium animalis* subsp. *lactis* BB12 was used as an anti-inflammatory reference [52].

### 2.5. In Vitro Epithelial Barrier Model

The human colon epithelial cell line Caco-2 clone TC7 [53] was grown at 37 °C with 10% CO_2_ in Dulbecco’s Modified Eagle Medium (DMEM, Life Technologies, Grand Island, NY, USA) supplemented with 5% of heat-inactivated fetal calf serum, 1% of non-essential amino acids, 2 mM glutamine (Gibco). 100 U/mL penicillin and 100 µg/mL streptomycin. For the permeability assay, cells were grown on 12-well Transwell^®^ insert filter (polycarbonate membrane with 0.4 µm pore size, 12 mm diameters; Costar, Corning Life Science, Kennebunk, ME, USA) at a density of 10^5^ cells per cm^2^, as previously described [46]. The medium was changed every two days until day 17 when optimal trans-epithelial resistance was reached (TEER 1800 W/cm^2^). The measures of TEER were performed using a millicell-ERS (Electrical Resistance System; Millipore, Billerica, MA, USA). Cell monolayers were then incubated for 30 min in a fresh DMEM medium. To test strains, bacteria were added in the apical compartment at a bacteria-to-cell ratio of 10:1. After 30 min, hydrogen peroxide (100 µM H_2_O_2_) was added in both apical and basal compartments. TEER was measured just before H_2_O_2_ addition (T0) and every 30 min up to 120 min. Three different experiments were performed including duplicates of each condition.

### 2.6. Enteroendocrine Cell Line and GLP-1 Induction

The intestinal neuroendocrine murine cell line STC-1 (kindly provided by Dr. Benoit Cudennec Institut Charles Viollette, Lille, France) was grown at 37 °C under 5% CO_2_ in DMEM (Life Technologies), supplemented with 10% of fetal calf serum (Dutscher, Brumath, France), 5 mM L-glutamine and 100 µg/mL of streptomycin and penicillin. Cells were seeded in 12 well plates at 200,000 cells/well, grown for 72 h, washed twice with PBS, and resuspended in 400 µL of 20 mM Hepes/20 mM Tris pH 7.4 buffer containing 140 mM NaCl, 4.5 mM KCl, 1.2 mM CaCl_2_, 1.2 mM MgCl_2_, 10 mM glucose. Cells were subsequently stimulated with the bacteria (10 µL) at a bacteria-to-cell ratio of 10:1 or with butyrate (10 mM final) as a positive control for 8 h at 37 °C under 5% CO_2_. The supernatants were then harvested, centrifuged (10 min at 8000× *g*), and stored at −20 °C. Quantification of active GLP-1 was performed using the V-Plex system and MESO QuickPlex SQ 120 (Meso Scale Diagnostics, Rockville, MD, USA).

### 2.7. Statistical Analysis

Statistical significance was determined using non-parametric Kruskal–Wallis followed by the Dunnett test (FDR method of Benjamini and Hochberg). Data with *p* values ≤ 0.05 were considered too significant.

## 3. Results

### 3.1. Strain-Dependent Survival to Gastric Stress

**Figure 1:**
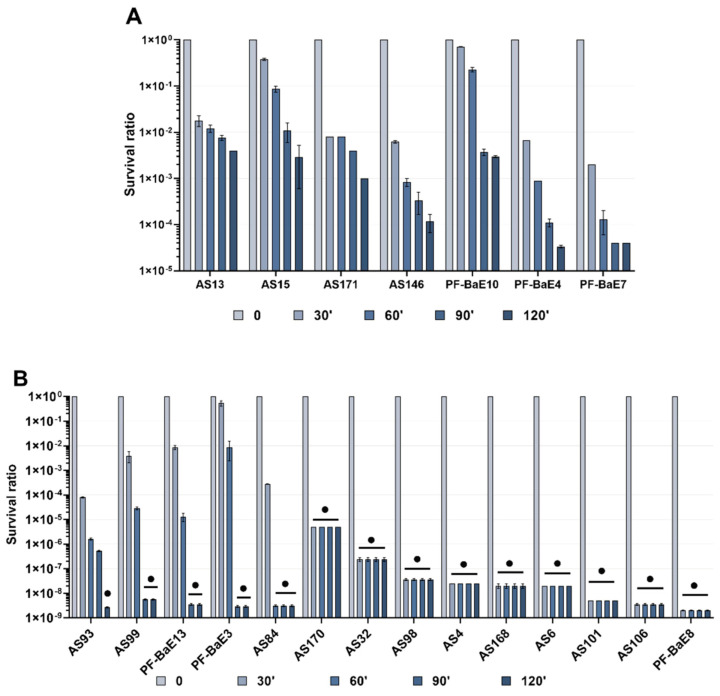
Bacterial survival to gastric stress at different timepoints. The survival is shown as the ratio of the colony-forming units (CFU/mL) at a given time point on the CFU/mL at T0. Data result from three independent experiments for each strain ± SEM. (**A**) Most tolerant strains partially surviving 120 min of gastric stress, (**B**) Sensitive strains. ● indicates the samples in which the CFU/mL reached the detection threshold which differs between strains as it depends on the initial bacterial cell concentration.

As the ability to survive gastric conditions is a criterion largely requested in the selection of probiotics, we investigated the tolerance of our selected set of 21 bacteria isolated from stools of neonates or adults (Table 1) to this stress. The test conditions (pH = 3 and pepsin) mimicked those encountered by bacteria in a stomach without food bolus [50]. In this harsh stress condition, none of the strains totally survived to 120 min incubation but we observed marked differences between strains (Figure 1). We distinguished two groups based on the bacterial survival to stress condition. The first group includes the 7 most tolerant strains to the stress: at 120 min of incubation, they remained viable albeit with a 2 to 4.5 log decrease in viability (Figure 1A). These gastric-stress tolerant bacteria were *B. intestinihominis* AS13, *B. vulgatus* AS15 and PF-Ba10, *B. ovatus* AS171, *B. xylanisolvens* AS146, *B. fragilis* PF-BaE4, and *P. distasonis* PF-BaE7. The second group consists of the other bacterial strains which were more sensitive to the stress as no colonies were obtained at 120 min or even before. CFU was not obtained (i) after 120 min of incubation for *P. distasonis* AS93, (ii) at 90 min and 120 min of incubation for *B. xylanisolvens* AS99, *B. uniformis* PF-BaE13, *B. thetaiotaomicron* AS84, *B. caccae* PF-BaE3, or (iii) from 30 min to 120 min of incubation for *A. soehngenii* AS170, *B. obeum* AS32, *B. massiliensis* AS98, *L. saburreum* AS4, *D. formicigenerans* AS168, *R. intestinalis* AS6, *B. coprocola* AS101, *P. merdae* AS106, *B. uniformis* PF-BaE8 (Figure 1B).

Of note, strains belonging to the same species exhibited different survival abilities for *B. xylanisolvens* AS146 and AS99, *P. distasonis* PF-BaE7 and AS93, and *B. uniformis* PF-BaE13 and PF-BaE8 while only the two *B. vulgatus* AS15 and PF-BaE10 exhibited similar survival.

### 3.2. Production of SCFA by the Selected Bacterial Strains

**Figure 2:**
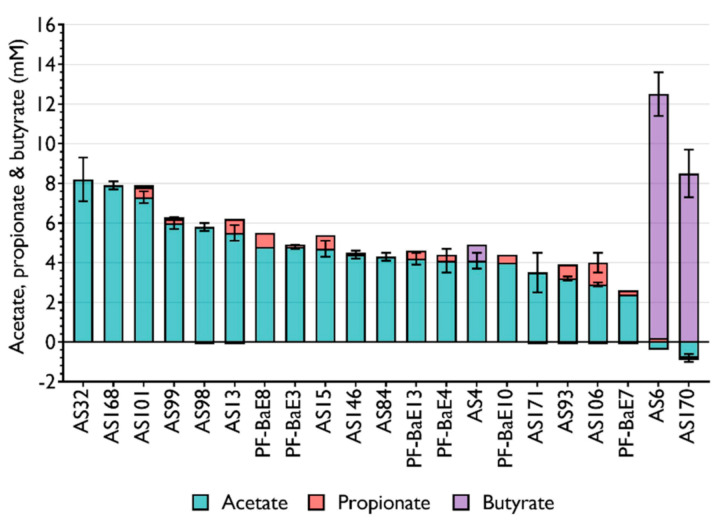
Production of acetate, butyrate, and propionate in the supernatant of bacterial cultures reported in mM for 1 OD_600nm_ unit of bacterial culture. Data are expressed as the means of 2 independent experiments ± SEM.

Knowing that SCFA are major players in gastrointestinal health, and immune and metabolic homeostasis [54], we evaluated their production by the selected bacteria during their growth in standard conditions e.g., in culture media which were not adjusted to optimize the SCFA production by each strain (Figure 2). *R. intestinalis* AS6 (12.3 mM for 1 OD_600nm_ unit) and *A. soehngenii* AS170 (8.5 mM per OD unit) produced a large amount of butyrate while they slightly consumed the amounts of acetate present in the media. The other strains mainly produced acetate at concentration reaching 8.2 mM per 1 OD unit for *R. obeum* AS32, the best producer. Of note, 14 strains were also able to produce a low amount of propionate.

### 3.3. Ability of the Strains to Strengthen the Epithelial Barrier

**Figure 3:**
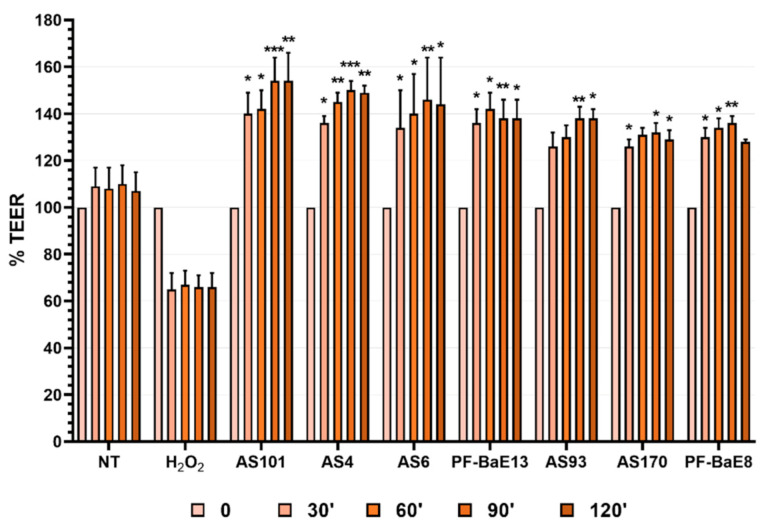
Strains preventing H_2_O_2_-induced paracellular permeability in vitro. Caco-2 cell monolayers were put in contact with the selected bacteria (MOI 10:1) and then sensitized with H_2_O_2_ at T0 (100 µM). NT corresponds to the untreated cells control and H_2_O_2_ to the cells only treated with oxygen peroxide. The %TEER was calculated as follows: TEER at Time X/TEER at T0 × 100. For each condition, data were expressed as the means of 3 independent experiments ± SEM. Statistical analysis refers to the comparison of cells treated with bacteria and H_2_O_2_ versus the H_2_O_2_ control. * *p* < 0.05; ** *p* < 0.01, *** *p* < 0.001.

First, we verified that the bacteria were catalase-negative i.e., were not able to degrade H_2_O_2_ when put in contact with it (data not shown). Second, the ability of the selected strains to modulate the H_2_O_2_-induced increase in paracellular permeability was evaluated using an in vitro epithelial barrier model as previously described [40,46,47]. As expected, the addition of H_2_O_2_ reduced the TransEpithelial Electrical Resistance (TEER) indicating an increased permeability (Figure 3, H_2_O_2_) reflecting the sensitization of the cell monolayers in the control. Seven strains significantly protected the cell monolayer from the H_2_O_2_-induced TEER decrease overtime: *B. coprocola* AS101, *L. saburreum* AS4, *R. intestinalis* AS6, *P. distasonis* AS93, *B. uniformis* PF-BaE13, *B. uniformis* PF-BaE8, and *A. soehngenii* AS170 (Figure 3, *p* < 0.05 to 0.001). Of note, few strains also transiently improved the TEER compared to the H_2_O_2_ treated control: *B. fragilis* PF-BaE4, *B. xylanisolvens* AS99, *D. formicigenerans* AS168, and *B. vulgatus* PF-BaE10 (Figure S1).

### 3.4. Immunomodulation by the Selected Strains

**Figure 4:**
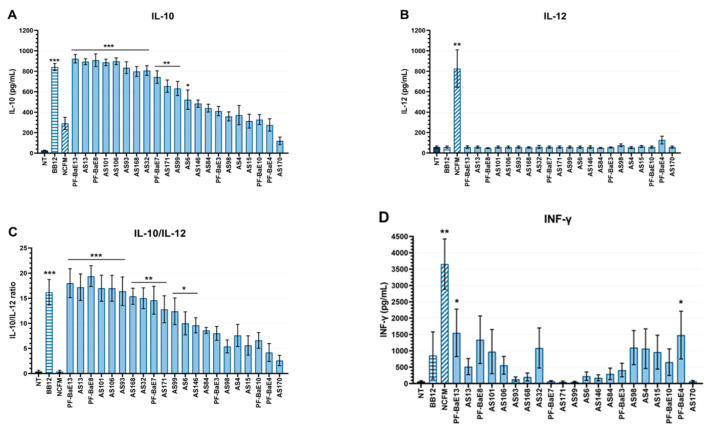
In vitro immunomodulatory profiles after PBMCs stimulation by the selected bacteria. PBMCs were stimulated 24 h with the selected strains or two reference strains: *L. acidophilus* NCFM (NCFM) and *B. animalis* subsp. *lactis* BB12 (BB12) at a ratio of 10:1 (bacteria:cells). The cytokine levels were measured by ELISA in the various samples and the untreated cells (NT). Data represent means ± SEM of 5 independent donors. Levels of (**A**) IL-10, (**B**) IL-12, and (**D**) IFNγ were measured in the supernatants. (**C**) IL-10/IL-12 ratios. * *p* < 0.05, ** *p* < 0.01; *** *p* < 0.001 in comparison with untreated cells.

The immunomodulatory capacities of the strains were evaluated through the release of the anti-inflammatory cytokine IL-10 (Figure 4A) or of the pro-Th1 IL-12 (Figure 4B) and IFN-γ (Figure 4D) cytokines after in vitro stimulation of human immune cells (PBMCs) by the selected bacteria.

Twelve of the 21 selected strains induced significant production of IL-10 (*p* < 0.05 to 0.001) in comparison to untreated cells, albeit in a strain-dependent manner, with values ranging between 485 ± 35 pg/mL to 924 ± 41 pg/mL. Six strains induced a level comparable (from 836 to 924 pg/mL) to the anti-inflammatory reference strain *B. animalis* subsp. *lactis* BB12 (842 ± 35 pg/mL, *p* < 0.001). Similar to the BB12 positive control, none of the selected strains induced a significant production of IL-12 (<100 pg/mL) while the pro-Th1 *L. acidophilus* NCFM reference strain induced high IL-12 levels (827 ± 13 pg/mL, *p* < 0.01) in comparison to untreated cells. The IL-10/IL-12 ratio (Figure 4C) confirmed the high anti-inflammatory potential of 10 strains (*p* < 0.01 to 0.001): *B. uniformis* PF-BaE13, *B. intestinihominis* AS13, *B. uniformis* PF-BaE8, *B. coprocola* AS101, *P. merdae* AS106, *P. distasonis* AS93, *D. formicigenerans* AS168, *B. obeum* AS32, *P. distasonis* PF-BaE7, *B. ovatus* AS171. In addition, *B. xylanisolvens* AS99, *R. intestinalis* AS6, and *B. xylanisolvens* AS146 also displayed a significant effect although to a lower extent (*p* < 0.05). The ability of the tested strains to induce the release of IFN-γ varied but remained insignificant for the vast majority of selected bacteria apart from *B. uniformis* PF-BaE13 and *B. vulgatus* PF-BaE4 which led to a low but significant IFN-γ production (*p* < 0.05). It can be compared with the pro-inflammatory *L. acidophilus* NCFM and anti-inflammatory *B. animalis* subsp. *lactis* BB12 controls which led to the production of 3664 ± 770 pg/mL (*p* < 0.01) and 862 ± 722 pg/mL of IFN-γ, respectively.

### 3.5. Ability of the Strains to Induce the Secretion of GLP-1

**Figure 5:**
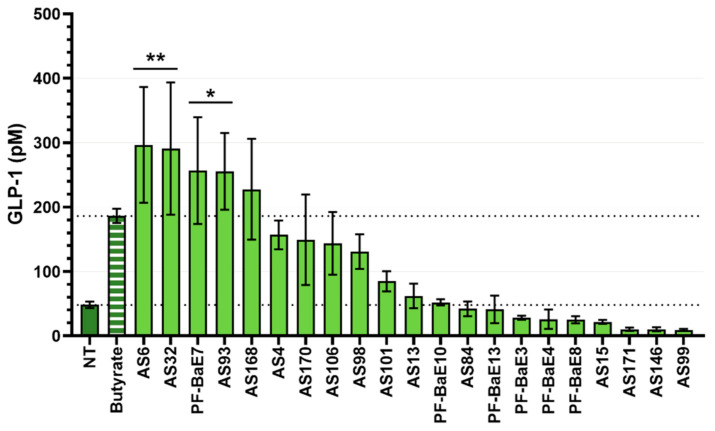
Production of GLP-1 in the supernatants of STC-1 cells stimulated by the selected strains. Cells were stimulated 8 h with the bacteria (MOI 10:1) or with butyrate (10 mM) as a positive control (Butyrate) then GLP-1 concentration was measured by Multiplex. * *p* < 0.05; ** *p* < 0.01 in comparison with untreated cells (NT).

The ability of the strains to stimulate the secretion of the gut peptide GLP-1 was evaluated using the murine cell line STC-1, displaying a phenotype of intestinal endocrine L-cells [55]. Four strains, *R. intestinalis* AS6 (*p* < 0.001), *B. obeum* AS32 (*p* < 0.01), *P. distasonis* PF-BaE7, and *P. distasonis* AS93 (*p* < 0.05) induced significant production of GLP-1 in comparison with the control of the untreated cell and to a higher level than the production induced by butyrate, the positive control. In addition, *D. formicigenerans* AS168 increased the release of GLP-1 in comparison with unstimulated cells although without statistical significance (Figure 5). Note that the test was performed without inhibiting the bacterial proteases which may contribute to GLP1 degradation.

### 3.6. Combination of Beneficial Properties in Next Generation Probiotic Candidates

**Figure 6:**
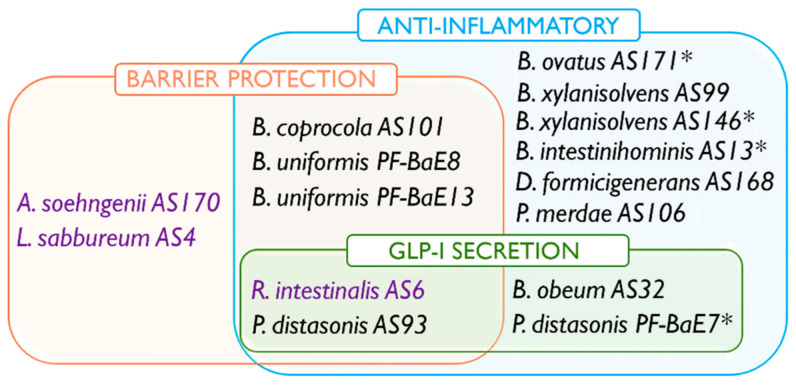
Diagram summarizing the beneficial properties of candidates as next-generation probiotics. Out of the 21 selected strains, 15 exhibited at least one statistically significant effect in the in vitro models including 7 strains that combined beneficial activities. Strains able to produce butyrate are highlighted in purple; note that AS4 produced only a limited amount of butyrate in the tested conditions. * indicates the bacterial strains which survived 2 h of gastric stress.

The integration of data generated in this study highlighted seven strains that are combining two or three of the probiotic properties tested in vitro (Figure 6).

Two strains, *P. distasonis* AS93 and *R. intestinalis* AS6 combined the three investigated functional activities (strengthening of the epithelial barrier, anti-inflammatory profile, and induction of GLP-1). The three strains *B. coprocola* AS101, *B. uniformis* PF-BaE8, and *B. uniformis* PF-BaE13 combined an anti-inflammatory profile and the ability to improve the epithelial barrier, while two strains *B. obeum* AS32 and *P. distasonis* PF-BaE7 combined an anti-inflammatory profile and the ability to induce GLP-1.

The three strains *R. intestinalis* AS6, *A. soehngenii* AS170, and *L. sabbureum* AS4 producing significant butyrate amounts were all able to strengthen the epithelial barrier, however, there was no correlation with the other functional activities. The bacterial survival to gastric stress was strain-dependent and only *P. distasonis* PF-BaE7 among the seven strains combining several probiotic functions was tolerant to that stress condition. However, we previously showed that adding sodium bicarbonate to the bacterial suspensions to neutralize the gastric pH, improved the strain survival during the in vitro stress assay (data not shown) and could also allow significant protection against intestinal inflammation after in vivo administration of gastric-stress sensitive strains [40].

## 4. Discussion

The gut microbiota is widely recognized to play a prominent role in health and disease and its altered composition and function are linked to the development of many chronic diseases, such as obesity and IBD. Therefore, manipulation of the dysbiotic gut microbiota towards a more balanced microbial community is currently under extensive study. Large screening of bifidobacteria and lactobacilli allowed the identification of several strains with health-promoting properties in pre-clinical models, however, these traditional probiotics showed marginal positive effects in clinical trials [43,44]. A recent study highlighted the transient persistence of these bacteria with permissive or resistant individuals, according to the level of colonization [56]. This suggests that at best, the probiotic effects persist during a short period of time [57]. Moreover, these types of probiotics, since their long history of safe use and their non-disease-specific claims, are generally delivered as food supplements. They are hardly considered for clinical applications and globally were not approved by regulatory authorities such as the European Food Safety Authority (EFSA) in Europe [58,59] or the Food Drug Administration (FDA) in the US [60].

The next-generation probiotics (NGP) or live biotherapeutic products (LBP) derived from the gut microbiota are developed as new preventive and therapeutic tools against diseases. Being natural gut commensals, the NGP may be better adapted to the gastrointestinal tract and the cross-talk with the host cells than food-derived probiotics [61]. Several strains enriched in healthy individuals in comparison to patients have been selected as live biotherapeutics (LBP) candidates. *F. prausnitzii*, a species depleted in the microbiome of Crohn’s patients appeared as a promising LBP for the management of this disease [31,35]. *A. muciniphila*, a mucin degrading bacterium resides in the mucus layer. Its abundance is strongly decreased in obese and type 2 diabetic subjects [23,34,62] and it has been shown to counteract high-fat diet-induced obesity, insulin resistance and type 2 diabetes in mice. More specifically, treatments of mice with this bacteria decreased metabolic endotoxemia and adipose tissue inflammation by improving intestinal mucosal barrier function at different levels [34]. However, the challenge of using these commensal gut bacteria as LBP relies mainly on their extreme sensitivity to O_2_ [62]. Therefore, the identification of new candidates possibly with a higher growth rate and tolerance to oxygen is still awaited for microbiota-targeted therapy. In this context, we screened a collection of bacterial strains, all members of the human gut microbiota using different in vitro models highlighting their functional abilities.

To the best of current knowledge, leaky gut and inflammation are key factors for the onset and development of numerous chronic diseases. The ability to protect the integrity of the epithelial barrier or to exhibit anti-inflammatory activities appears as key features for LBP. We observed that seven selected strains significantly maintained the epithelial barrier integrity over time in comparison with the control of H_2_O_2_-sensitized cells while several additional strains also exhibited a trend to barrier strengthening without robust statistical difference. Twelve strains significantly induced the production of the anti-inflammatory cytokine IL-10 after stimulation of human immune cells without noticeable effect on IL-12 production, leading to a robust anti-inflammatory profile (IL-10/IL12 ratio). Note, however, that *B. uniformis* PF-BaE13 stimulated the IFN-γ release to a low but significant level. This will have to be further examined for instance in the preclinical model before concluding on the anti-inflammatory potential of that strain. GLP-1 plays an important role not only in the control of food intake but also in the control of gut barrier function, glucose, and energy homeostasis, and, consequently insulin sensitivity [63]. Four strains (AS6, AS32, PF-BaE7, and AS93) were able to significantly induce the release of GLP-1 from the enteroendocrine cell line STC-1. Altogether, our results revealed a high prevalence of health beneficial activities among the 21 tested gut bacteria with 71.4% of strains positive in at least one of the three in vitro tests. This observation suggests the involvement of these strains in active cross-talk with host cells.

Strikingly 33% of the strains possessed multiple putative beneficial activities (Figure 6). Three strains combined an anti-inflammatory IL10/IL12 profile and the ability to protect the epithelial barrier. Two strains, *P. distasonis* PF-BaE7 and *B. obeum* AS32, combined an anti-inflammatory profile and a stimulation GLP1 secretion. *R. intestinalis* AS6 and *P. distasonis* AS93 interestingly combined the three tested activities: barrier strengthening, anti-inflammatory profile, and stimulation of GLP1 secretion. It is noteworthy that among the 4 pairs of strains assigned to given species, 3 showed similar phenotypes: *B. uniformis* PF-Ba8 and PF-BaE13 appeared both as an anti-inflammatory and protecting the barrier; *B. xylanisolvens* AS146 and AS99 were both anti-inflammatory while *B. vulgatus* AS15 and PF-BaE10 did not possess the tested activities. The two *P. distasonis* AS93 and PF-BaE7 are discordant for the barrier restoration activity but shared an anti-inflammatory profile and the ability to stimulate GLP-1 production.

*B. coprocola* AS101 and *B. uniformis* PF-BaE8 and PF-BaE13 which combined anti-inflammatory profile and barrier protection abilities together with *R. intestinalis* AS6 and *P. distasonis* AS93 which showed the 3 probiotic activities are highlighted by our work as appealing NGP candidates to target intestinal inflammation. Indeed, based on the in vitro barrier and immunomodulation tests, we were previously able to identify *P. distasonis* strains including AS93, with the ability to alleviate intestinal inflammation in a murine model of colitis [40]. In the present study, we included two of these *P. distasonis* strains to complete their functional characterization with their effect on GLP1 production. Interestingly in our previous work, *P. distasonis* PF-BaE7 was not protective in a murine model of TNBS-induced colitis in contrast to *P. distasonis* AS93. We propose that the difference between these two strains in terms of barrier strengthening made the difference in the colitis model.

The *P. distasonis* AS93 and *R. intestinalis* AS6 and possibly *B. obeum* AS32 and *P. distasonis* PF-BaE7 strains are interesting candidates as NGPs in the context of obesity. Obesity is indeed associated with impaired endocrine metabolism, together with leaky gut, low-grade inflammation, and gut microbiota dysbiosis. *Parabacteroides* is one of the major genera of the human core gut microbiota [64]. In agreement with our in vitro data, the potential of a *P. distasonis* strain as NGP against obesity has been recently reported [41]. Interestingly, live but not heat-killed *P. distasonis* CGMCC 1.30169 reduced weight gain, improved glucose homeostasis, and obesity-associated dysfunction [41]. A link between *Roseburia* spp. and gut health has been reported, with anti-inflammatory activities as well as beneficial metabolic effects, primarily through the production of short-chain fatty acids, especially butyrate, suggesting a potential use as LBP in many chronic diseases [65]. Indeed, we highlighted *R. intestinalis* AS6 as a butyrate producer, together with the highest capacity to induce the release of GLP-1, in comparison to other strains. This strain appears as a potential candidate against obesity.

Obesity has also been linked with differential abundance of *Bacteroides*, *Parabacteroides*, *Blautia*, *Alistipes*, *Romboutsia*, and *Roseburia*. Most of those genera are recognized to regulate the host immune system and some have been shown to alleviate obesity [65]. Large discrepancies and heterogeneity among the gut microbiota associated with obesity and metabolic diseases in general are observed according to the cohorts, with divergent results for phylum, family, genus, and species. The discrepancies in the gut microbial signatures observed among studies could be explained by the difference in age, ethnicity, diet, circadian rhythm, microbiome sequencing methods, and other variables [66]. Therefore several hurdles remain to ascertain the implication of specific taxa as a causal link to the development of obesity or other chronic diseases or conversely to beneficial effects [67]. Decreased *Bacteroidetes* to *Firmicutes* ratio remains a significant risk indicator [18], however different studies reported a positive association between some *Bacteroides* species i.e., *B. fragilis*, and obesity [68]. Interestingly several experimental studies highlighted the beneficial impact of several *Bacteroides* strains, such as *B. uniformis* CECT771 [61], *B. acidifaciens* ICM10556 [39] which led to a positive impact on weight gain and other parameters associated with obesity, by acting through different mechanisms. *B*. *fragilis* HCK-B3 and *B*. *ovatus* ELH-B2 have also been proposed as novel strains to alleviate intestinal inflammation [69]. In our study, 3 *Bacteroides, B. uniformis* PF-BaE8 and PF-BaE13, and *B. coprocola* AS101 exhibited interesting functional properties and could also be proposed as NGP candidates. One mechanism by which gut microbes can communicate and initiate beneficial effects is linked to their capacity to produce short-chain fatty acids (SCFAs), mostly acetate, propionate, and butyrate [70]. In the tested conditions which were not optimized for SCFA production, two strains, *R. intestinalis* AS6 and *A. soehngenii* AS170 produced butyrate (12.3 mM and 8.5 mM for 1 OD_600nm_ unit), respectively while the other strains mainly produced acetate at varying concentrations (from 2.4 to 8.2 mM per OD_600nm_ unit). Even if the 2 butyrate-producing strains were able to strengthen the epithelial barrier, no correlation between the capacity of the strains to produce SCFA in vitro with functional activities was observed. However, it is noteworthy that the in vitro conditions do not reflect the in vivo situation in which the commensal strains could favor the production of SCFA through the degradation of fibers but also by the mechanism of cross-feeding among the gut microbiota. The effects we measured were assessed with live bacteria. Preliminary data performed with some of the strains indicated that similar effects, and in some cases increased effects, could be obtained with pasteurized (heat-killed at 70 °C) bacteria. Plovier et al., have indeed reported that pasteurized *A. muciniphila* could exhibit similar or even better abilities to prevent diet-induced obesity in mice and have highlighted the potential role of a membrane protein [71]. This opens the way to counteract the oxygen sensitivity of some strains and to use derived metabolites as postbiotics. It would be therefore interesting to further decipher the mechanisms involved in the strain’s effects and identify the bacterial factors involved. Therefore, the influence of the gut microbiome by itself on the overall immune and metabolic functions still needs further investigation.

## 5. Conclusions

Although harnessing the microbiome of healthy versus disease state highlighted potential candidates to be used as NGP, identification of novel health-associated gut bacteria to be used as LBP has to be extended. This will not only allow better insight into the functionality of the different species and strains but also extend the number of interesting candidates for the development of personalized probiotic approaches taking better into account individual host variations and specific responses [40,72]. Our in vitro screening approach provided crucial clues to highlight the health beneficial abilities of several strains with promising use as novel NGP candidates, notably in the management of IBD and obesity. Further experiments to establish the innocuity of the strains of interest, to investigate in appropriate preclinical models, their efficacy, and to decipher the underlying mechanisms are underway.

## Figures and Tables

**Table 1:** Bacterial strains and growth media.

Strain	Species	Growth Medium	Origin
AS4	*Lachnoanaerobaculum saburreum*	BHI-YS	Healthy adult feces
AS6	*Roseburia intestinalis*	BHI-YS	Healthy adult feces
AS13	*Barnesiella intestinihominis*	BHI-YS	Healthy adult feces
AS15	*Bacteroides vulgatus*	BHI-YS	Newborn fecal samples
AS32	*Blautia obeum*	BHI-YS	Healthy adult feces
AS84	*Bacteroides thetaiotaomicron*	BHI-YS	Healthy adult feces
AS93	*Parabacteroides distasonis*	BHI-YS	Healthy adult feces
AS98	*Bacteroides massiliensis*	BHI-YS	Healthy adult feces
AS99	*Bacteroides xylanisolvens*	BHI-YS	Healthy adult feces
AS101	*Bacteroides coprocola*	BHI-YS	Healthy adult feces
AS106	*Parabacteroides merdae*	BHI-YS	Healthy adult feces
AS146	*Bacteroides xylanisolvens*	BHI-YS	Healthy adult feces
AS168	*Dorea formicigenerans*	BHI-YS	Healthy adult feces
AS170	*Anaerobutyricum soehngenii* DSMZ17630	M104S	Healthy infant feces
AS171	*Bacteroides ovatus*	BHI-YS	Healthy adult feces
PF-BaE3	*Bacteroides caccae*	BHI-YS	Newborn fecal samples
PF-BaE4	*Bacteroides fragilis*	BHI-YS	Newborn fecal samples
PF-BaE7	*Parabacteroides distasonis*	BHI-YS	Newborn fecal samples
PF-BaE8	*Bacteroides uniformis*	BHI-YS	Newborn fecal samples
PF-BaE10	*Bacteroides vulgatus*	BHI-YS	Newborn fecal samples
PF-BaE13	*Bacteroides uniformis*	BHI-YS	Newborn fecal samples
NCFM	*Lactobacillus acidophilus*	MRS	DuPont™ Danisco-Madison, USA
BB12	*Bifidobacterium animalis* subsp. *lactis*	MRS Cystein	Dietary origin (Chr Hansen, Hoesholm, Denmark)

## Data Availability

Data is contained within the article or supplementary materials.

## Supplementary Materials

The following are available online at https://www.mdpi.com/2076-2607/9/3/565/s1, Figure S1: Impact of the other selected strains on H_2_O_2_-induced paracellular permeability in vitro. * *p* < 0.05
