# Supplementary figures and images for: Identification of New Potential Biotherapeutics from Human Gut Microbiota-Derived Bacteria
Source: Microorganisms. 2021 Mar 9;9(3):565. doi: 10.3390/microorganisms9030565 (PMC7998412; doi:10.3390/microorganisms9030565)

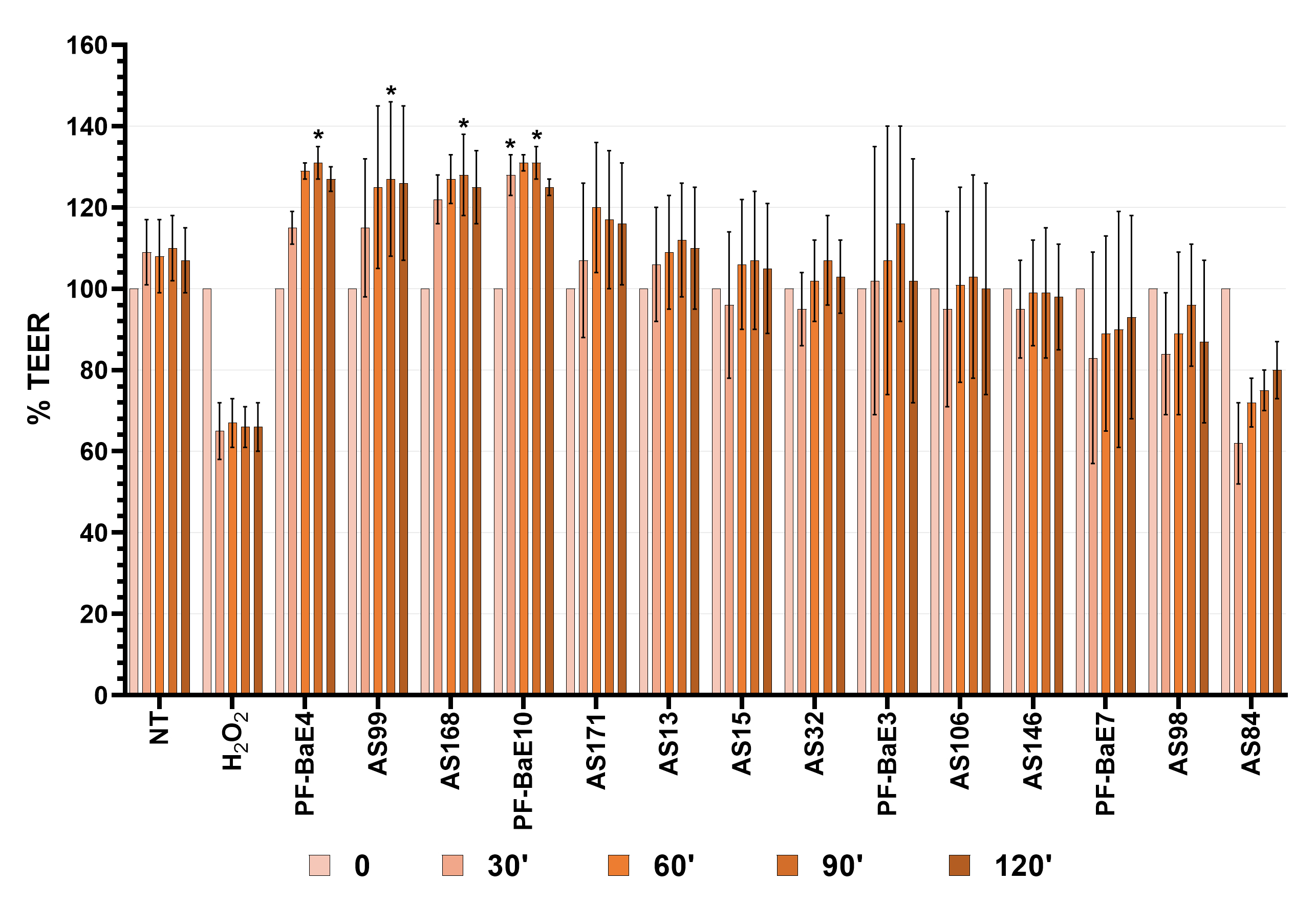

Supplement: Supplementary file 1 [file microorganisms-09-00565-s001.zip › microorganisms-1116316-supplementary.jpg]
